# Cognitive reserve and adaptive resilience: a conceptual analysis of a cross-species framework for neuropsychology and cognitive aging

**DOI:** 10.3389/fpsyg.2026.1948103

**Published:** 2026-09-22

**Authors:** Yunier Broche-Pérez, Diego D. Díaz-Guerra, Marena de la C. Hernández-Lugo, Carlos Ramos-Galarza

**Affiliations:** 1 Department of Behavior Analysis, Prisma Behavioral Center, West Palm Beach, FL, United States; 2 Departamento de Psicología, Facultad de Ciencias Sociales, Universidad Central “Marta Abreu” de Las Villas, Santa Clara, Cuba; 3 Departamento de Psicología, Facultad de Ciencias Sociales, Centro de Bienestar Universitario, Universidad Central “Marta Abreu” de Las Villas, Santa Clara, Cuba; 4 Facultad de Psicología, Centro de Investigación ESTEC, Universidad Tecnológica Indoamérica, Quito, Ecuador

**Keywords:** animal behavior, behavioral flexibility, cognitive aging, cognitive reserve, compensation, environmental enrichment, evolutionary adaptation, resilience

## Abstract

Why do some older adults maintain effective cognitive functioning despite substantial age-related brain change or neuropathology, whereas others with comparable neural burden show marked impairment? Related questions arise in comparative research, where organisms exposed to similar biological or environmental challenges may show markedly different adaptation patterns. Although these phenomena have traditionally been examined in separate literatures, they converge on a broader problem: how living systems maintain, recover, or reorganize function under challenge. This article develops a cross-species conceptual framework linking research on adaptive resilience with contemporary models of cognitive reserve and cognitive aging. The proposed contribution is not to redefine cognitive reserve as dynamic or lifespan-based, features already recognized in current reserve theory, but to identify a set of candidate mechanisms through which reserve-related capacities may be developed and expressed. We organize these mechanisms across four interacting domains: biological regulation, behavioral flexibility, social regulation, and environmental engagement and enrichment. These processes are hypothesized to contribute, across development and aging, to the capacity to adapt when biological or environmental demands increase. A fifth domain, functional preservation and recovery, represents the outcome of these processes rather than cognitive reserve itself. In humans, preserved cognition despite neural burden may therefore be understood as a cognitive-resilience phenotype to which cognitive reserve, brain maintenance, compensation, and related mechanisms can contribute. Nonhuman animal models do not possess cognitive reserve in its human clinical sense, but they provide experimental access to potentially conserved mechanisms involving stress regulation, flexible responding, social buffering, learning, and experience-dependent plasticity. Cognitive reserve remains the primary translational focus of the present framework because of its central role in cognitive aging and dementia research, while recognizing that related mechanisms may also operate in affective, motor, behavioral, and neuroautonomic domains. The framework generates testable predictions about how measurable adaptive processes may complement traditional reserve proxies and improve understanding of individual differences in cognitive aging.

## 1 Introduction

Why do some organisms continue to function adaptively under conditions that impair others? This question appears in very different areas of research. In nonhuman primate studies, young squirrel monkeys exposed to brief, mild, intermittent early stress later showed stronger socioemotional and neuroendocrine resistance to novel challenges, as well as improved prefrontal-dependent response inhibition (Parker et al., 2005, 2004, 2006). In cognitive aging research, some older adults maintain better cognitive performance than would be expected from their degree of brain aging or neuropathology, a pattern that has been central to the development of the cognitive reserve construct (Stern, 2002, 2012). Longitudinal clinicopathologic studies further show that cognitively stimulating activity across the lifespan is associated with slower late-life cognitive decline, even after accounting for common neuropathologic conditions (Wilson et al., 2013). At first glance, these findings belong to separate domains: one to comparative neuroendocrinology and the other to dementia epidemiology. Yet both point to the same broader problem: how adaptive systems preserve, recover, or reorganize function in the face of challenge.

Resilience is often used to describe this capacity, but the term does not always mean the same thing across fields. In developmental science, resilience is often referred to as positive adaptation in the context of significant adversity (Masten, 2001). In stress neuroscience, it has been linked to the regulation of physiological responses to challenge and recovery from allostatic disruption (McEwen, 1998). In animal research, resilience is usually inferred from observable indicators, such as sustained exploration, adaptive coping, recovery after perturbation, and the preservation of functional behavior over time, rather than from subjective reports (Scheffer et al., 2018). In cognitive aging, a related idea is that cognitive performance is better than expected given the observed degree of age-related brain change, injury, or neuropathology (Stern et al., 2020).

This diversity has been scientifically productive, but it has also created a fragmented language. Researchers studying resilience in rodents or nonhuman primates, resilience in children exposed to adversity, and resilience in older adults at risk for dementia often work in parallel rather than in conversation. As a result, shared mechanisms, stress-response regulation, behavioral flexibility, learning from experience, social buffering, environmental engagement, and compensatory strategy use are frequently described under different names.

This manuscript argues that these literatures can be brought into closer conceptual alignment. The central proposal is that resilience reflects a family of evolutionarily conserved adaptive mechanisms that support the maintenance, recovery, or reorganization of functional behavior after biological or environmental challenge. These mechanisms are not expressed identically across species, nor should they be measured in the same way. However, across organisms, they involve overlapping regulatory and behavioral capacities: modulating stress responses, adjusting behavior when environmental contingencies change, using social resources, learning from prior challenge, and engaging with complex environments.

In humans, the gradual development of these capacities across the lifespan may help explain what cognitive-aging researchers have called cognitive reserve. From this perspective, cognitive reserve is neither solely a neural endowment nor simply the accumulation of education, occupational attainment, or intellectual activity. Rather, histories of regulating, adapting, compensating, and remaining engaged in the face of changing demands may represent processes that contribute to the development or expression of cognitive reserve.

A terminological distinction is therefore necessary. In the aging literature, cognitive reserve refers to individual differences in cognitive processes that allow some people to perform better than expected given the degree of age-related brain change, injury, or neuropathology (Stern, 2002; Stern et al., 2020). Brain reserve refers more directly to structural or quantitative neural resources, whereas brain maintenance refers to the relative preservation of brain integrity over time (Stern et al., 2020). Recent frameworks have proposed resilience as an overarching construct encompassing reserve- and maintenance-related processes, while encouraging operational definitions applicable across human and model-system research (Kremen et al., 2022; Stern et al., 2023). The present manuscript follows this approach: cognitive reserve is retained as a human clinical and cognitive-aging construct, whereas resilience is used as the broader cross-species adaptive concept.

Accordingly, the bridge proposed here is one of mechanism rather than identity. Animal models of resilience can help isolate regulatory, flexible, and compensatory processes under controlled conditions. Human studies of cognitive reserve can show how similar capacities accumulate across development, education, work, social life, and aging. Connecting these levels of analysis may clarify how reserve is built, how it is expressed, and why it varies so strongly across individuals.

The novelty of the present framework is not the claim that resilience or cognitive reserve are dynamic, nor the proposal of a lifespan perspective; both ideas are already established in contemporary reserve and resilience research. The proposed advance is instead mechanistic and comparative. We organize a set of measurable adaptive processes (biological regulation, behavioral flexibility, social regulation, and environmental engagement) and hypothesize that these processes help shape the development and expression of human reserve-related capacities and, under neural challenge, the preservation or reorganization of function. The framework therefore shifts the level of analysis from broad labels and indirect proxies toward candidate mechanisms that can be examined experimentally across species, while preserving the conceptual distinctions among reserve, maintenance, compensation, and resilience outcomes (Table 1, Figure 1).

**Table 1:** Conceptual positioning of the proposed adaptive-resilience framework relative to reserve and resilience constructs.

Construct	Primary focus	Temporal/ functional emphasis	Typical operationalization	Relation to proposed framework	References
Cognitive reserve	Better-than-expected cognition given life-course brain change/injury/disease; mechanisms may be molecular, cellular, network, or cognitive.	Active; influenced across the lifespan.	Education/IQ or other proxies; moderation; cognitive/neural experimental approaches.	Primary human translational target. Proposed cross-species processes are candidate contributors to its development/expression, not a redefinition of CR.	(Stern et al., 2020, 2023)
Cognitive resilience	Preserved/better-than-expected cognition despite adverse brain factors.	Outcome/phenotype under challenge; often longitudinal.	Moderation or residual/discrepancy methods, depending on framework.	Closest mapping to Level 5 cognitive outcome. Should not be conflated with CR.	(Kremen et al., 2022; Bocancea et al., 2021; Elman et al., 2022)
Brain reserve	Neurobiological capital/status.	Resource level at a point in time; not active adaptation.	Brain volume, neuronal/synaptic or related structural features.	Potential contributor to preserved function, but conceptually distinct from behavioral flexibility or regulation.	(Stern et al., 2023)
Brain maintenance	Relative preservation of neural resources/pathology over time.	Longitudinal preservation.	Change in brain markers linked to preserved cognition.	Parallel protective pathway; not the same as behavioral adaptation.	(Stern et al., 2023)
Compensation	Cognition-enhancing recruitment of neural resources under high demand or age-related loss.	Real-time/task-related response to demand.	Functional activation and performance patterns; selection, upregulation, reorganization.	One plausible mechanism through which adaptive behavior is expressed under neural challenge.	(Cabeza et al., 2018)
Affective reserve	Capacity to regulate affective states/dispositions across adversity, pathology, or aging.	Affective adaptation across the lifespan.	Emotion/motivation regulation, coping, social support; operationalization still developing.	Complementary domain-specific reserve; supports the claim that reserve need not be cognitively exclusive.	(Di Rosa, 2024)
Motor reserve	Preserved motor function despite disease-related neural burden.	Motor-system adaptation/compensation.	Physical activity and motor/neural measures; definitions still being standardized.	Complementary domain-specific reserve demonstrating outcome-specific reserve mechanisms.	(Giustiniani and Quartarone, 2024)
Behavioral reserve	Less severe behavioral disturbance than expected for neural burden, studied especially in bvFTD.	Behavioral phenotype under disease burden.	Behavior-neuroimaging discrepancy/residual-type approaches.	Shows that reserve-related phenomena can be expressed outside conventional cognitive outcomes.	(Kim et al., 2022)
Neuroautonomic Adaptive Resilience (NAR)	Dynamic self-regulatory coordination between central and autonomic nervous-system processes supporting adaptation to cognitive and emotional stressors.	Dynamic, real-time regulation across stress phases and lifespan.	HRV/EDA or related autonomic profiles.	Dynamic resilience mechanism positioned within biological regulation. It may interact with cognitive reserve, maintenance, and compensation but does not constitute a reserve mechanism itself.	(Fusi et al., 2026)
Proposed adaptive-resilience framework	Cross-species mechanisms linking life-course experience to adaptive functioning under challenge.	Developmental + dynamic; mechanisms and outcomes explicitly separated.	Biological regulation, behavioral flexibility, social regulation, environmental engagement, and functional preservation/recovery.	Adds a comparative mechanistic layer. Levels 1–4 are candidate pathways; Level 5 is functional outcome/resilience phenotype.	Present manuscript

**Figure 1:**
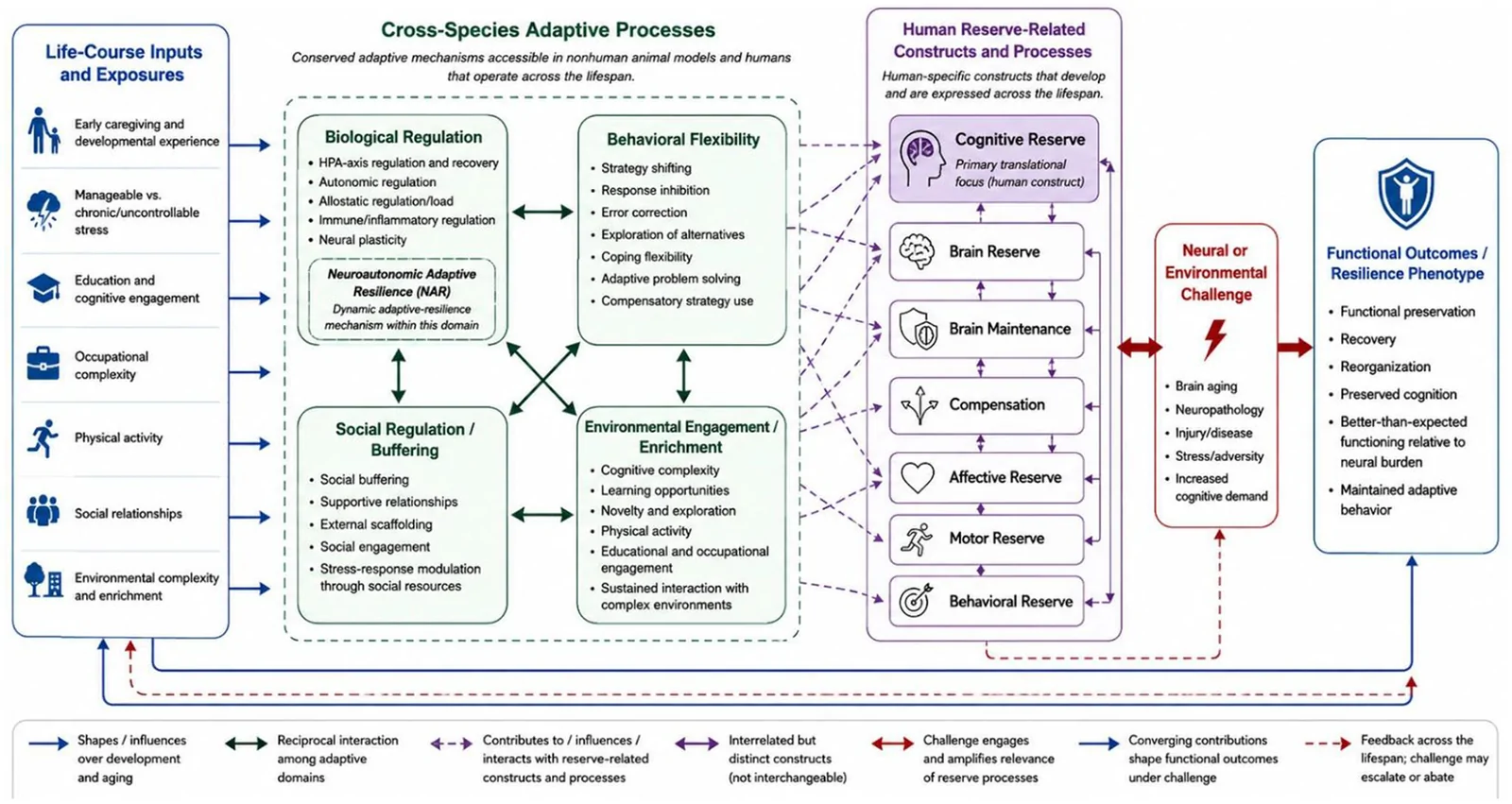
Conceptual positioning of the adaptive-resilience framework. Life-course exposures shape biological, behavioral, social, and environmental adaptive processes that can be studied across species. In humans, these processes are hypothesized to contribute to the development or expression of reserve-related mechanisms, including cognitive reserve, and to functional resilience when neural or environmental challenges occur. The figure distinguishes candidate mechanisms **(middle)** from reserve constructs (human layer) and functional outcomes **(right)**, thereby avoiding equating cognitive reserve with resilience.

This argument has theoretical and empirical implications. Theoretically, it links animal stress research, developmental science, behavioral neuroscience, and the cognitive neuroscience of aging within a shared adaptive framework. Empirically, it predicts that behavioral indicators of resilience, such as coping flexibility, adaptive strategy use, social engagement, environmental exploration, and stress recovery, should help explain cognitive aging trajectories above and beyond structural brain measures and traditional reserve proxies such as education or occupational attainment. It further predicts that these behavioral capacities may mediate part of the protective effect currently attributed to cognitive reserve.

## 2 Resilience as a dynamic adaptive process

### 2.1 The problem with trait-based definitions

A central tension in resilience research concerns whether resilience should be understood as a relatively stable individual trait or as a dynamic adaptive process (Denckla et al., 2020). The trait-based view has intuitive appeal. Some organisms and individuals do show relatively consistent differences in temperament, behavioral inhibition, stress reactivity, emotional regulation, and exploratory behavior (Boyce and Ellis, 2005; Koolhaas et al., 1999). From this perspective, it may seem reasonable to describe some individuals as “resilient” and others as “vulnerable.” However, this approach creates a conceptual problem: it can make resilience appear to be something an organism possesses or lacks, rather than something that emerges through the interaction between biological systems, developmental history, environmental demands, and available resources.

For this reason, contemporary resilience science has increasingly moved toward process-oriented and systems-based definitions. Rutter explicitly described resilience as a dynamic concept, emphasizing that it must be inferred from patterns of adaptation following adversity rather than treated as a fixed attribute of the person (Rutter, 2012). Similarly, interdisciplinary accounts have defined resilience in terms of adaptive functioning over time after adversity, while also emphasizing that resilience depends on context, developmental timing, social resources, and the nature of the challenge itself (Southwick et al., 2014). A process view, therefore, allows resilience to vary across developmental periods, stressor types, environmental contexts, and available behavioral and social supports. It also strengthens resilience through experience, learning, environmental enrichment, social connection, and the gradual development of regulatory capacities. Conversely, resilience may be weakened when those same resources are degraded, overloaded, or absent.

This view is also consistent with research on resilience trajectories following loss, trauma, and other potentially adverse events. Bonanno (2004) proposed that many individuals exposed to potentially traumatic events show a relatively stable pattern of healthy functioning rather than chronic dysfunction or delayed disturbance. Later work further distinguished resilience from recovery, chronic distress, and delayed symptom trajectories, showing that adaptation after adversity is heterogeneous rather than uniform (Bonanno and Mancini, 2008; Bonanno et al., 2011). Importantly, resilience in this framework does not mean the complete absence of distress. Rather, it refers to the preservation or restoration of adaptive functioning through regulatory, cognitive, emotional, social, and behavioral mechanisms.

This process-oriented framing is especially useful for a cross-species account of resilience. In humans, resilience can be studied through self-report, clinical interview, behavioral observation, physiological measures, and longitudinal outcomes (Connor and Davidson, 2003; Ewart et al., 2002; O'Donohue et al., 2021). In nonhuman animals, it must be inferred from observable patterns of behavior and biological regulation, such as sustained exploration, recovery after perturbation, social approach, flexible problem-solving, and preservation of reward-seeking behavior (Beery and Kaufer, 2015; Krishnan et al., 2007; Lea et al., 2020). In both cases, resilience is best understood not as a static label, but as a pattern of adaptive organization over time.

### 2.2 Biological grounding: allostasis and allostatic load

The biological foundation most relevant to a process-oriented account of resilience is the allostasis and allostatic load framework. Allostasis refers to the capacity of biological systems to maintain stability through change by adjusting physiological activity in response to actual or anticipated environmental demands (Pfaltz and Schnyder, 2023). Rather than assuming a fixed regulatory set point, the allostatic model emphasizes flexible coordination among multiple systems, including the hypothalamic-pituitary-adrenal axis, the autonomic nervous system, metabolic processes, immune activity, and inflammatory responses (McEwen, 1998, 2000; McEwen and Stellar, 1993).

This flexibility is adaptive. Stress mediators such as cortisol, catecholamines, and inflammatory signaling molecules support short-term mobilization, enabling the organism to respond to threats, uncertainty, novelty, or increased environmental demands (Charmandari et al., 2005; Godoy et al., 2018; Russell and Lightman, 2019). However, the same systems that protect the organism in the short term may become harmful when they are activated repeatedly, remain elevated for too long, or fail to shut down efficiently after the challenge has passed. This cumulative cost has been described as allostatic load: the biological “wear and tear” associated with repeated or chronic adaptation to stressors (Juster et al., 2010; McEwen, 1998; McEwen and Stellar, 1993).

From this perspective, resilience is not the absence or suppression of stress responding. A resilient organism does not fail to react. Rather, resilience involves the capacity to mount an appropriate response, regulate its intensity, and recover efficiently once the demand has ended. The timing, magnitude, and reversibility of the stress response are therefore central. Successful regulation may preserve adaptive functioning and support future flexibility, whereas repeated dysregulation may gradually compromise the same systems that originally served a protective function.

This biological framing has direct relevance for cognitive aging. The brain is not only a regulator of allostatic processes but also a target of their cumulative effects. Chronic exposure to stress mediators has been linked to structural and functional changes in brain systems involved in memory, executive control, emotional regulation, and behavioral flexibility, including the hippocampus and prefrontal cortex (McEwen, 2000, 2002). Empirical studies in aging samples have also associated higher allostatic load with poorer later-life brain health and cognitive functioning (Booth et al., 2015). A systematic and meta-analytic review further found that higher allostatic load was associated with poorer global cognition and executive function in adults. However, associations with memory were less consistent (D'Amico et al., 2020).

The allostatic model, therefore, helps connect resilience and cognitive reserve. If resilience depends partly on efficient biological regulation across repeated challenges, then lifelong patterns of stress regulation, recovery, coping, and environmental engagement may influence the development and expression of reserve. In this sense, resilience is not only a psychological or behavioral construct. It is also a biological process through which adaptive regulation may protect cognitive function over time.

### 2.3 A working definition

In the present framework, resilience is defined as a dynamic, adaptive process by which an organism maintains, recovers, or reorganizes functional behavior after exposure to adversity, stress, threat, deprivation, or biological challenge. This process emerges through ongoing interactions among biological regulation, behavioral flexibility, learning history, social resources, and environmental engagement. In this sense, resilience is not treated as a fixed possession of the individual, but as a pattern of adaptive functioning that unfolds over time and in relation to changing environmental demands (Masten, 2001; Rutter, 2012; Southwick et al., 2014).

This definition is intentionally broad enough to apply to humans and nonhuman animals, while remaining specific enough to be operationalized in empirical research. In humans, resilience may be assessed through behavioral, psychological, physiological, and longitudinal indicators of adaptation. In nonhuman animals, it must be inferred from observable patterns such as maintained exploration, flexible responding, social approach, recovery after perturbation, and preservation of goal-directed or reward-seeking behavior (Scheffer et al., 2018). Across species, the emphasis is therefore placed not on subjective experience alone, but on the organization of behavior after challenge.

This working definition also clarifies the role of behavior in the proposed framework. Biological regulation may support resilience, and social or environmental resources may strengthen it. Still, resilience is ultimately expressed through what the organism can do: continue functioning, adapt to changing conditions, recover after disruption, or develop new strategies when previous patterns are no longer effective. This behavioral emphasis makes resilience a useful bridge between animal models, developmental science, stress biology, and cognitive aging research.

## 3 Evidence from animal research

### 3.1 Why animal models matter

Animal research is essential for a cross-species account of resilience because it allows investigators to study adaptation under conditions that cannot be created experimentally in humans. In animal models, stressors can be introduced with greater control over timing, intensity, duration, predictability, and controllability. Developmental periods can be followed prospectively, and behavioral outcomes can be measured alongside neural, endocrine, immune, molecular, and epigenetic indicators. This makes it possible to test not only whether an organism adapts after challenge, but also how that adaptation is produced.

These models are especially valuable because many of the proposed protective factors in human resilience research, such as maternal care, environmental enrichment, social housing, physical activity, stress controllability, and early experience, can be experimentally manipulated in animals. Classic work in rodents, for example, has shown that natural variation in maternal care is associated with enduring differences in offspring stress responses, and that these effects may involve changes in hippocampal glucocorticoid receptor expression and epigenetic regulation (Francis et al., 1999; Weaver et al., 2004). Similarly, environmental enrichment has been used to examine how complex sensory, motor, cognitive, and social stimulation shapes neural plasticity, learning, and behavioral adaptation (van Praag et al., 2000). Stress controllability paradigms have also shown that the ability to exert behavioral control over an aversive event can alter subsequent stress responses, implicating prefrontal regulatory mechanisms in resilience-like outcomes (Maier et al., 2006; Maier and Watkins, 2005).

The value of animal research is not that animals reproduce human resilience in a complete or literal way. They do not capture the full subjective, cultural, linguistic, or autobiographical dimensions of human adaptation. Rather, their value lies in isolating conserved biological and behavioral mechanisms under controlled conditions. Contemporary translational models of resilience emphasize this point: animal studies are most informative when they focus on mechanisms, individual differences, developmental timing, and behavioral profiles rather than assuming a simple one-to-one correspondence with human psychiatric or cognitive outcomes (Daskalakis et al., 2013; Richter-Levin et al., 2019).

For the present framework, animal models matter because they allow resilience to be studied as an observable pattern of adaptation. Across species, resilience can be inferred from sustained exploration, flexible responding, recovery of reward-seeking, social approach, problem-solving, and physiological regulation after challenge. These outcomes provide a behavioral bridge between experimental animal research and human studies of cognitive reserve. If cognitive reserve is partly built through repeated engagement with demanding and enriched environments, then animal models can help identify the regulatory and plasticity mechanisms through which such engagement becomes biologically and behaviorally protective.

### 3.2 Rodents: social defeat, susceptibility, and resilience

Chronic social defeat paradigms in rodents are among the most influential experimental models for studying individual differences in stress adaptation. In these paradigms, mice or rats are exposed to repeated social subordination. They are later assessed across behavioral domains such as social interaction, exploration, sucrose preference, anxiety-like behavior, and immobility in forced-swim or tail-suspension tests. Standardized chronic social defeat protocols have shown that repeated exposure to an aggressive conspecific can produce enduring social avoidance and other depression-like behavioral changes in a subset of animals (Golden et al., 2011). However, one of the most important findings from this literature is that animals exposed to the same stressor do not respond uniformly.

In a foundational study, Krishnan et al. (2007) showed that an inbred population of mice exposed to chronic social defeat could be separated into susceptible and unsusceptible subgroups, with differences across behavioral, physiological, molecular, and electrophysiological domains. This finding is central to the present argument because it shows that resilience-like outcomes are not explained simply by lower stress exposure. Rather, they reflect different patterns of adaptation after comparable challenges. Importantly, resilient animals are not merely “less affected” versions of susceptible animals. Their profiles may involve active compensatory processes within neural systems involved in reward, motivation, and stress regulation (Ashokan et al., 2016; Krishnan et al., 2007).

This distinction is conceptually important. If resilience were only the absence of pathology, then susceptible and resilient animals would be expected to fall along a single continuum of impairment. Instead, rodent studies suggest that resilience can involve active biological organization. For example, experimental work on chronic social defeat has implicated mesolimbic dopamine circuitry, prefrontal regulatory pathways, and molecular adaptations that distinguish susceptibility from resistance to stress (Quessy et al., 2021; Zhao et al., 2021). These findings support a process-oriented interpretation of resilience: adaptive functioning after adversity is not passive preservation, but an organized pattern of regulation, compensation, and behavioral adjustment.

Early social experience is one of the clearest examples of how resilience-related systems can be shaped developmentally. Meaney's (2001) work on maternal care in rats showed that naturally occurring variation in licking, grooming, and arched-back nursing was associated with long-term differences in offspring HPA-axis regulation, behavioral fearfulness, and cognitive outcomes. Cross-fostering studies further supported the role of the postnatal caregiving environment by showing that offspring stress responses were shaped by the maternal behavior they received, not only by inherited biological background (Francis et al., 1999). Later work identified an epigenetic pathway through which maternal behavior altered hippocampal glucocorticoid receptor expression, providing a mechanism by which early caregiving experience could become biologically embedded (Weaver et al., 2004).

Environmental enrichment studies extend this developmental logic beyond caregiving. Enriched environments typically combine increased sensory, motor, cognitive, and social stimulation through access to novel objects, complex spatial layouts, running wheels, and social housing. In rodents, such environments have been associated with changes in exploratory behavior, anxiety-like response, synaptic plasticity, hippocampal neurogenesis, and cognitive performance (Nithianantharajah and Hannan, 2006). Importantly, enrichment can also alter later responses to stress. For example, environmental enrichment before social defeat has been shown to confer stress resiliency through mechanisms involving the infralimbic cortex, a prefrontal region implicated in behavioral regulation and adaptive control (Lehmann and Herkenham, 2011).

These rodent findings are directly relevant to the cognitive reserve argument. They show that environmental experience can shape the biological systems that support later adaptation. Maternal care, enrichment, controllability, and social context do not simply reduce exposure to adversity; they modify how the organism responds when adversity occurs. By analogy, human cognitive reserve may emerge not only from accumulated intellectual resources but also from a long history of behavioral engagement with complex, demanding, and socially embedded environments. In both cases, the protective effect lies less in avoiding challenge than in developing systems capable of regulating, compensating, and adapting when challenge appears.

### 3.3 Nonhuman primates: stress inoculation and regulatory development

Nonhuman primate studies are especially relevant to a cross-species account of resilience because primates share with humans prolonged developmental immaturity, complex social organization, and highly flexible behavioral repertoires for coping, exploration, and social regulation (Phillips et al., 2014). These features make primate models particularly useful for examining how early experience shapes later stress regulation, executive control, and adaptive behavior.

The stress inoculation model developed in squirrel monkeys provides one of the clearest examples. In this paradigm, young monkeys were exposed to brief, repeated separations from their mothers during development. These separations were stressful, but they were also time-limited, intermittent, and followed by reunion and recovery. Compared with non-inoculated controls, stress-inoculated monkeys later showed reduced behavioral and neuroendocrine responses to novel stressors, suggesting stronger socioemotional and HPA-axis regulation (Lyons and Parker, 2007; Parker et al., 2004).

Subsequent work also showed that mild early stress enhanced prefrontal-dependent response inhibition, supporting the idea that manageable challenge may strengthen neural systems involved in cognitive control and adaptive regulation (Parker et al., 2005). These findings are also supported by evidence that stress inoculation was associated with changes in ventromedial prefrontal cortical development (Katz et al., 2009). Publisher records describe the 2004 study as prospective evidence that moderately stressful early experience strengthened socioemotional and neuroendocrine resistance to later stressors.

A key strength of this literature is that it does not present adversity as inherently beneficial. Parker and Maestripieri (2011) emphasized that early stress can lead to either vulnerability or resilience, depending on features such as timing, severity, duration, frequency, developmental period, ecological validity, and sensory modality. Their review is important because it avoids a simplistic “more stress makes stronger organisms” interpretation. Instead, it suggests that early challenges may support resilience only under specific conditions: when it is moderate, manageable, intermittent, and embedded within a context that allows recovery and social buffering. The same review explicitly argues that early-life stress effects are not simply linear and that primate models help clarify which features of stress exposure contribute to vulnerability vs. resilience.

The role of controllability is especially important. Experimental work on stressor controllability has shown that behavioral control over an aversive event changes both immediate and later stress responses. Maier and Watkins argued that controllable stress engages ventromedial prefrontal mechanisms that inhibit stress-responsive brainstem and limbic systems, reducing the impact of stress and promoting later resistance to new stressors (Maier and Watkins, 2010). This finding provides a mechanistic bridge between stress inoculation and cognitive reserve. Manageable challenge may strengthen prefrontal systems involved in top-down regulation, flexible responding, response inhibition, and compensatory control. In contrast, severe, chronic, or uncontrollable stress is more likely to increase allostatic load and undermine regulatory capacity.

This distinction is central to the present framework. Challenge may become resilience-building when it provides opportunities for regulation, problem-solving, recovery, and successful adaptation. It may become biologically costly when it overwhelms the organism's regulatory capacity or occurs without control, predictability, or social buffering. Lyons and Parker described this developmental process as a cascade linking stress inoculation, arousal regulation, and resilience, emphasizing that experiences that are challenging but not overwhelming may promote later adaptive regulation (Lyons et al., 2009). This framing aligns closely with the broader resilience literature in humans, where related processes have been discussed under concepts such as steeling, stress inoculation, ordinary adaptive systems, and differential susceptibility (Belsky and Pluess, 2009; Bonanno and Mancini, 2008; Masten, 2001).

The primate evidence therefore adds biological plausibility to the proposed connection between resilience and cognitive reserve. It suggests that adaptive functioning is not built by avoiding all challenges, but by repeated encounters with demands that are difficult enough to require regulation and flexible responding, yet not so overwhelming as to produce chronic dysregulation. In humans, lifelong engagement with educational, occupational, social, and cognitively complex environments may operate comparably: not merely by adding intellectual “resources,” but by repeatedly exercising the regulatory and compensatory systems through which reserve is expressed.

### 3.4 Dogs: applied behavioral resilience

Dogs offer a valuable translational perspective because they are a domesticated species whose development, welfare, and behavior are closely shaped by human environments. Companion and working dogs face repeated challenges, including separation, rehoming, veterinary procedures, training demands, encounters with unfamiliar people, social conflict, novelty, and changes in routine. Individual differences in fearfulness, anxiety, sociability, behavioral flexibility, and recovery after stress are readily observable in dogs and have direct implications for welfare, training success, and behavioral health.

The canine literature is especially useful for the present framework because it brings resilience closer to applied behavior. Tiira argued that resilience is a meaningful and underdeveloped construct in canine behavioral science and identified several candidate protective factors, including early enrichment, appropriate socialization, positive human–dog relationships, predictability, opportunities for behavioral control, and social support from humans or conspecifics (Tiira, 2019). This review explicitly connected canine resilience to broader comparative work, making dogs a useful bridge between laboratory models of stress adaptation and real-world behavioral outcomes.

Recent work has also begun to operationalize resilience more directly in pet dogs. (Mackay et al., 2023a,b) developed the Lincoln Canine Adaptability and Resilience Scale, a proxy-report instrument designed to assess owner-perceived resilience-related behavior in dogs. The resulting scale included two main components: adaptability or behavioral flexibility, and perseverance. Although this approach is closer to a trait-level assessment than to the dynamic process emphasized in the present manuscript, it is still important because it demonstrates that resilience-related constructs can be translated into measurable behavioral dimensions in a nonhuman species. The authors also noted the need for future validation using independent behavioral and physiological correlates, which aligns with the broader argument that resilience should be assessed through multiple levels of evidence.

Developmental and environmental studies in dogs further support the idea that resilience-related behavior is shaped by experience. Reviews of puppy socialization indicate that early exposure to people, animals, objects, handling, and varied environments may reduce later fearfulness and improve adaptive behavior when exposures are structured, gradual, and positive rather than overwhelming (Howell et al., 2015). In a large survey study of nearly 6,000 pet dogs, Puurunen et al. (2020) found that social fearfulness was associated with poor socialization during puppyhood, infrequent participation in training and activities, urban environment, and several demographic and breed-related factors. This pattern is consistent with the central claim of the present framework: adaptive functioning reflects an interaction between biological predispositions, developmental experience, environmental exposure, and opportunities for behavioral engagement.

The applied relevance of canine resilience is also evident in working-dog research. Fear and anxiety can compromise welfare and performance, and reviews of working-dog development emphasize the importance of temperament, learning history, environmental influences, and structured exposure in reducing maladaptive fear responses (Rooney et al., 2016). This is important because working dogs provide a practical example of resilience as functional behavior under demand. Success is not simply the absence of fear; it requires recovery from stress, flexible responding, sustained task engagement, responsiveness to handlers, and the ability to perform in complex and changing environments.

Dogs, therefore, contribute something distinctive to a cross-species model of resilience. Unlike many laboratory models, canine resilience can be studied in naturalistic, socially embedded settings where adaptive behavior has immediate consequences for welfare and functioning. At the same time, dogs do not require verbal reports to infer resilience. Their behavior can be observed through approach, avoidance, exploration, recovery, training persistence, social referencing, and adjustment to environmental change. For this reason, the dog literature strengthens the behavioral bridge between research on animal resilience and human cognitive reserve. It shows how resilience can be operationalized as an applied pattern of adaptation: not simply whether an organism has been protected from stress, but whether it can continue to learn, engage, recover, and function when the environment becomes demanding.

### 3.5 Broader biological models

Resilience is not a psychological concept alone. A broader biological perspective treats resilience as a property of living systems that can be observed across multiple levels of organization. Thorogood et al. (2023) argued for a multilevel framework of biological resilience, defining it as the set of processes that allow components across biological levels, from genes and genomes to cells, organisms, populations, communities, and ecosystems, to resist disturbance or recover after perturbation. This perspective is important for the present manuscript because it places resilience within a general adaptive framework rather than limiting it to human psychological functioning.

At this broader biological level, resilience involves two related but distinct capacities: resistance and recovery. Resistance refers to the degree to which a system maintains function during disturbance, whereas recovery refers to the degree and speed with which function returns after perturbation (Reed et al., 2024). These concepts can be applied to neural systems, stress physiology, behavior, populations, or ecosystems, even though the specific measurements differ across levels. This provides a useful conceptual bridge for the present argument: cognitive reserve in humans may be one specialized expression of a more general biological principle, namely the capacity to preserve or reorganize function when challenged.

Molecular and physiological approaches further reinforce this point. Basile et al. (2020) reviewed how animal resilience can be studied through omics approaches, including genomic, transcriptomic, epigenomic, proteomic, and metabolomic methods. Their review emphasized that resilience is not a single inherited capacity, but a dynamic process involving environmental stressors, epigenetic modulation, neural plasticity, physiological regulation, and behavioral adaptation. This is important because it shows that resilience-related behavior is supported by coordinated biological processes across multiple levels, rather than by a single mechanism or isolated trait.

This biological depth strengthens the cross-species framework proposed here. Behavioral resilience may be observed at the surface through exploration, recovery, social engagement, flexible responding, and sustained function. However, these behavioral outcomes are supported by regulatory processes operating at neural, endocrine, immune, molecular, and ecological levels. The broader biological literature, therefore, helps clarify why resilience should not be reduced either to a personality trait or to a purely psychological outcome. It is better understood as a multilevel adaptive process through which living systems respond to challenge while attempting to preserve, recover, or reorganize function.

For the cognitive reserve argument, this broader view is especially useful. If resilience operates across levels of biological organization, then human cognitive reserve can be interpreted as a late-life manifestation of adaptive regulation accumulated over the lifespan. Education, occupation, social engagement, physical activity, and cognitively demanding environments may not simply add “resources” to the brain. They may repeatedly engage biological and behavioral systems of regulation, plasticity, compensation, and recovery. In this sense, cognitive reserve can be framed as a human, cognitive-aging expression of a more general biological capacity for adaptive function under challenge.

### 3.6 Model systems and the mechanistic study of reserve

Recent work has explicitly argued that model systems can contribute to the mechanistic study of cognitive reserve. The value of these models does not depend on assuming that animals possess human-like cognitive reserve in the full clinical or autobiographical sense. Rather, their value lies in allowing researchers to study how biological aging, neural change, and preserved cognitive function relate to one another under controlled longitudinal conditions.

McQuail et al. (2020) emphasized that animal models are especially useful for identifying early predictors of later cognitive vulnerability or resilience, characterizing molecular, cellular, and network-level mechanisms, and experimentally testing factors that are mostly correlational in human studies. Their review specifically framed model systems as tools for understanding how the relationship between brain structure, brain function, and cognition changes across the lifespan, and how some organisms maintain cognitive performance despite biological aging or neural vulnerability (McQuail et al., 2020).

This point strengthens the present framework. If cognitive reserve in humans is reflected in better-than-expected cognitive performance under conditions of brain aging, injury, or neuropathology, then animal aging models can help identify the mechanisms that underlie this preserved function. Longitudinal animal designs make it possible to examine whether enriched experience, stress regulation, physical activity, social housing, environmental complexity, or behavioral flexibility alters the relationship between brain change and cognitive outcome.

The translational value of animal models, therefore, lies not in assigning human clinical constructs directly to animals. It lies in experimentally isolating mechanisms that may later appear in humans as reserve-related cognitive resilience. These mechanisms may include synaptic plasticity, neurogenesis, network efficiency, inflammatory regulation, stress-response calibration, compensatory recruitment, and preserved learning capacity. In this sense, model systems allow cognitive reserve to be studied not only as a statistical discrepancy between pathology and performance, but also as a biological and behavioral process that can be traced across development, aging, and experience.

This approach is particularly important to the manuscript's broader argument. Animal studies of resilience show how organisms maintain, recover, or reorganize behavior after stress or challenge. Model-system studies of cognitive reserve extend this logic into aging by asking how organisms preserve cognitive function despite biological change. Together, these literatures suggest that resilience and reserve are not separate conceptual islands. They are related expressions of adaptive regulation: one emphasizing response to adversity or stress, and the other emphasizing preserved cognitive function under neural challenge.

## 4 Human psychological resilience

### 4.1 Developmental perspectives

In developmental science, resilience has been most influentially understood not as an exceptional quality, but as the result of ordinary adaptive systems operating under conditions of adversity. Masten's (2001) “ordinary magic” framework argued that children's resilience emerges from basic developmental processes such as secure attachment, effective caregiving, self-regulation, cognitive competence, problem-solving capacity, motivation to adapt, and support from families, schools, and communities. This view is important because it moves resilience away from the idea of rare psychological strength and toward a more developmental, systems-based account of adaptation.

This framework has several implications for the present argument. First, resilience is not a special capacity possessed only by unusually strong individuals. It emerges from the same biological, behavioral, cognitive, and social systems that support normative development. Second, resilience is multidetermined. No single protective factor is sufficient in all cases, and strengths in one system may sometimes compensate for vulnerabilities in another. Third, resilience is modifiable. If resilience depends on adaptive systems, then interventions that strengthen caregiving, self-regulation, learning opportunities, social support, and environmental stability may improve adaptation even in high-risk populations.

This developmental view is consistent with earlier critical work emphasizing that resilience should not be treated as a fixed trait or a global label. Luthar et al. (2000) argued that resilience refers to positive adaptation despite significant adversity and should be studied with careful attention to the nature of the risk, the domain of adaptation, and the developmental context in which adaptation occurs. In other words, a child may show resilience in one domain, such as academic functioning, while continuing to struggle in another, such as emotional regulation or peer relationships. Resilience is therefore better understood as a pattern of adaptation within specific contexts rather than as a general personal attribute.

Southwick et al. (2014) extended this analysis across biological, psychological, social, and cultural levels of explanation. Their interdisciplinary review emphasized that resilience cannot be adequately captured at any single level of analysis and that definitions vary depending on whether researchers focus on individuals, families, communities, neurobiology, trauma exposure, or cultural context. This reinforces a central point of the present manuscript: resilience is not a single mechanism but a coordinated adaptive process involving multiple interacting systems.

The context-specific nature of resilience is especially important. An individual may adapt well after bereavement but struggle under chronic economic hardship; another may function effectively under social stress but become vulnerable when exposed to illness, caregiving burden, or institutional instability. This variability should not be treated as a weakness of the resilience construct. Rather, it is one of its defining features. Resilience depends on the match between the challenge, the organism's regulatory capacities, the available social and environmental supports, and the developmental timing of exposure.

From the perspective of cognitive reserve, developmental resilience provides a crucial foundation. If adaptive functioning is built through repeated interactions among regulation, learning, caregiving, social support, and environmental demand, then the capacities later described as reserve may begin long before old age. Cognitive reserve may therefore reflect not only formal education or occupational complexity, but also a broader developmental history of adaptive engagement with challenge. In this sense, human psychological resilience offers a developmental pathway through which behavioral flexibility, regulation, compensation, and environmental engagement can accumulate across the lifespan.

### 4.2 Coping, regulation, and behavioral flexibility

One of the most consistently supported mechanisms of human resilience is flexible regulation: the capacity to adjust coping, emotion-regulation, and problem-solving strategies in response to changing situational demands. Lazarus and Folkman's (1987) transactional model provided the theoretical foundation for this view by framing stress as a dynamic relation between environmental demands and perceived coping resources, rather than as a property of the event alone. From this perspective, coping involves ongoing cognitive and behavioral efforts to manage demands that are appraised as taxing, threatening, or exceeding available resources.

Effective coping is therefore not defined by the use of one universally adaptive strategy. Problem-focused coping may be useful when a stressor is controllable, whereas acceptance, reappraisal, emotion regulation, or support-seeking may be more adaptive when the situation cannot be changed directly. Contemporary work on regulatory flexibility makes this point explicit: adaptation depends not only on which strategies individuals possess but also on whether they can select, implement, monitor, and revise those strategies in response to situational demands (Bonanno and Burton, 2013).

Empirical evidence supports this flexible view of coping. A meta-analytic review found that coping flexibility was positively associated with psychological adjustment across stressful life changes, suggesting that individuals who can vary their coping responses and match strategies to contextual demands tend to show better adaptation (Cheng et al., 2014). This evidence is important for the present framework because it shows that resilience is not simply emotional toughness or the absence of distress. Rather, it involves the capacity to reorganize behavior when the environment changes.

Coping flexibility is also not best understood as a fixed endowment. Psychological flexibility, a closely related construct, has been described as a central component of mental health because it allows individuals to remain engaged with valued goals while adjusting to difficult thoughts, emotions, and changing circumstances (Kashdan and Rottenberg, 2010). Clinical and behavioral interventions, including acceptance and commitment therapy, explicitly target this capacity by strengthening flexible responding, values-based action, acceptance, and adaptive contact with present-moment contingencies (Hayes et al., 2006). This supports the developmental claim that coping flexibility can be cultivated through experience, intervention, and repeated practice rather than treated as a static trait.

The parallels with animal resilience research are direct. In rodents and other model systems, behavioral flexibility is commonly assessed through strategy shifting, reversal learning, inhibition of ineffective responses, exploration in the presence of novelty, and recovery of goal-directed behavior after stress. Environmental enrichment studies show that complex sensory, motor, cognitive, and social stimulation can modify neural plasticity, exploratory behavior, and learning-related outcomes (Nithianantharajah and Hannan, 2006). Thus, what human research describes as coping flexibility and what animal research describes as behavioral flexibility may represent related adaptive capacities expressed through species-appropriate forms.

This connection is central to the manuscript's broader argument. If resilience depends on the ability to regulate, shift strategies, recover from disruption, and remain engaged with meaningful or functional goals, then flexibility is one of the behavioral mechanisms through which resilience may translate into cognitive reserve. Across development, individuals repeatedly exposed to complex educational, occupational, social, and problem-solving demands may strengthen not only cognitive skills, but also the capacity to select and adjust strategies under changing conditions. Over time, this flexible behavioral repertoire may help explain why some individuals maintain cognitive performance despite age-related brain change or neuropathology.

#### 4.2.1 Shared neural mechanisms: the bridge between animal and human resilience

The claim that resilience is evolutionarily conserved rests on more than behavioral analogy. Although resilience is expressed differently across species, several neural systems provide mechanistic points of convergence between animal and human research. These systems do not prove that animal resilience and human psychological resilience are identical. Rather, they show that adaptive responses to stress, threat, novelty, and cognitive challenge are supported by partially shared biological architectures.

#### 4.2.2 Prefrontal-mediated executive control

The prefrontal cortex, particularly its connections with the amygdala, hippocampus, striatum, and brainstem stress-regulatory systems, plays a central role in behavioral flexibility, inhibitory control, emotional regulation, and adaptive decision-making. In animal models, stressor controllability engages medial and ventromedial prefrontal mechanisms that inhibit stress-responsive systems and reduce later vulnerability to new stressors (Maier and Watkins, 2010). In squirrel monkeys, mild early stress has been associated with improved prefrontal-dependent response inhibition and later stress regulation, suggesting that manageable challenge can strengthen systems involved in top-down control (Lyons and Parker, 2007; Parker et al., 2005). In humans, prefrontal recruitment during cognitive challenge is central to models of cognitive aging and compensation. The revised Scaffolding Theory of Aging and Cognition proposes that older adults may rely on compensatory neural scaffolding, including prefrontal recruitment, to maintain performance when primary networks become less efficient (Reuter-Lorenz and Park, 2014). Similarly, contemporary cognitive aging frameworks distinguish among maintenance, reserve, and compensation while emphasizing the role of flexible network recruitment in preserved cognition (Cabeza et al., 2018).

#### 4.2.3 Hippocampal plasticity and contextual learning

The hippocampus is highly sensitive to stress but also highly plastic. Chronic stress and elevated stress mediators can impair hippocampal structure and function, with consequences for memory, contextual learning, and cognitive flexibility (McEwen, 1998). Conversely, enriched environments and physical activity have been shown in rodents to influence hippocampal neurogenesis, synaptic plasticity, and learning-related outcomes (Kempermann et al., 1997; Nithianantharajah and Hannan, 2006; van Praag et al., 2000). In humans, aerobic exercise has been associated with increased anterior hippocampal volume and improved memory in older adults, providing a parallel example of experience-dependent plasticity in a reserve-relevant brain system (Erickson et al., 2011). This is directly relevant to the present framework because hippocampal function supports the kind of contextual and relational learning that allows organisms to update behavior, recognize when previous strategies no longer work, and adapt to changing environmental demands.

#### 4.2.4 HPA-axis regulation and glucocorticoid sensitivity

Efficient stress regulation requires not the absence of HPA-axis activation, but calibrated reactivity and effective recovery. In rodent models, maternal care shapes offspring stress reactivity and hippocampal glucocorticoid receptor expression, with long-term consequences for endocrine regulation and behavior (Meaney, 2001). Epigenetic work further showed that maternal behavior can alter hippocampal glucocorticoid receptor expression through DNA methylation-related mechanisms, offering a biological pathway by which early caregiving becomes embedded in stress-regulatory systems (Weaver et al., 2004). In humans, chronic stress and allostatic load have been linked to cumulative biological wear, including effects on brain systems involved in memory and executive control (McEwen, 1998). These findings suggest that lifelong stress regulation may be one mechanism through which resilience-related processes influence the neural systems that later support cognitive reserve.

#### 4.2.5 Social buffering

Across animal and human studies, the presence or support of an affiliative partner can reduce stress responses, including HPA-axis activation, although the strength and direction of this effect depend on relationship quality, developmental timing, prior experience, and species-specific social organization (Hennessy et al., 2009; Hostinar et al., 2014). This matters for cognitive reserve because social engagement is often treated as a proxy for cognitively stimulating experience. However, social relationships may contribute to reserve beyond what cognitive stimulation alone can. They may also reduce cumulative stress exposure, provide external scaffolding for coping and problem-solving, and support regulatory recovery after challenge. Thus, social engagement may contribute to cognitive reserve through at least two pathways: by exercising complex cognitive and communicative abilities, and by buffering biological stress systems that influence hippocampal and prefrontal aging.

Together, these mechanisms, prefrontal executive control, hippocampal plasticity, HPA-axis regulation, and social buffering, provide a biologically grounded bridge between animal resilience research and human cognitive reserve. They also identify specific measurement targets for future research. An individual's history of stress regulation, exposure to controllable challenge, quality of social buffering, integrity of prefrontal-hippocampal networks, and glucocorticoid sensitivity are all, in principle, assessable. These indicators may help clarify how resilience-related processes accumulate across development and become expressed in later life as cognitive reserve, compensatory capacity, or preserved cognitive function despite neural challenge.

## 5 Cognitive reserve as a human expression of adaptive resilience

### 5.1 The cognitive reserve concept

Cognitive reserve refers to individual differences in the ability to cope with brain aging, injury, or neuropathology. More specifically, it describes the observation that individuals with comparable levels of neural burden may differ substantially in the degree of cognitive impairment they show. The reserve concept emerged from clinical, neuropathological, and epidemiological observations showing a discrepancy between brain pathology and functional outcome. For example, early clinicopathological work identified individuals with preserved mental status despite numerous neocortical plaques at autopsy, suggesting that some brains may tolerate substantial Alzheimer-type pathology before clinical impairment becomes evident (Katzman et al., 1988). Later epidemiological work showed that lower educational and occupational attainment were associated with increased risk of incident Alzheimer's disease, supporting the idea that life experience may influence the clinical expression of neuropathology (Stern et al., 1994). The explanatory challenge was therefore to understand why similar levels of brain burden do not always produce similar cognitive outcomes.

Stern later formalized cognitive reserve as a construct that helps explain individual differences in susceptibility to age-related brain changes or Alzheimer-related pathology (Stern, 2012). In this view, reserve does not mean that pathology is absent. Rather, it means that some individuals are better able to maintain function in the presence of pathology, either by using cognitive strategies more efficiently, recruiting alternative networks, or relying on more flexible patterns of neural organization. This distinction is central to the present manuscript because it shifts cognitive reserve from a passive “buffer” toward an active adaptive capacity.

Stern et al. (2020) provided an important conceptual clarification by distinguishing cognitive reserve from brain reserve and brain maintenance. Brain reserve refers to relatively structural or quantitative characteristics, such as brain size, synaptic density, neuronal count, or other anatomical resources that may allow the brain to tolerate pathology before a clinical threshold is reached. Brain maintenance refers to the relative preservation of brain structure and function over time, reflecting genetic, biological, and lifestyle influences on the rate of neural aging. Cognitive reserve, in contrast, refers to individual differences in the flexible and efficient use of cognitive processes and neural networks that allow some people to perform better than expected despite brain aging, injury, or disease. In this sense, cognitive reserve is not simply a structural endowment. It is an active functional capacity.

Cabeza et al. (2018) further refined this distinction in cognitive-neuroscience terms by differentiating among maintenance, reserve, and compensation. Maintenance refers to the preservation of neural resources. Reserve refers to the efficiency, capacity, or flexibility with which existing neural resources support performance. Compensation refers to the recruitment of alternative neural networks or cognitive strategies when primary systems are no longer sufficient. These processes are not mutually exclusive. In an aging individual, preserved neural integrity, efficient use of existing networks, and compensatory recruitment may operate together. Compensation is especially relevant to the present framework because it parallels what animal research often refers to as behavioral flexibility, strategy shifting, or adaptive reorganization following disruption. Importantly, these mechanisms should also be distinguished from dynamic resilience processes such as NAR, which may influence the regulatory conditions under which reserve, maintenance, and compensation are expressed without constituting any of these mechanisms itself.

The revised Scaffolding Theory of Aging and Cognition, or STAC-r, provides a complementary neural account of compensation. Reuter-Lorenz and Park proposed that age-related neural decline can trigger the recruitment of supplementary neural circuitry, particularly involving frontal systems, to support cognitive performance when primary networks become less efficient (Reuter-Lorenz and Park, 2014). Importantly, STAC-r does not treat compensation as a purely late-life reaction to decline. It also emphasizes that life experiences, including cognitive engagement, physical activity, and social participation, may influence the availability and effectiveness of compensatory scaffolding. This interpretation aligns closely with the present framework: the same behavioral history that supports resilience across development may also strengthen the regulatory, cognitive, and compensatory capacities later expressed as cognitive reserve.

A more recent life-course formulation of the scaffolding framework makes this connection even clearer. Reuter-Lorenz and Park (2024) argued that STAC-r should be understood as a life-course model of neuroplasticity, in which enrichment and depletion factors shape both the level of cognitive functioning and the trajectories of cognitive change across the lifespan. This life-course emphasis is directly compatible with the present proposal. If compensatory scaffolding reflects adaptive neural recruitment in response to challenge, then the capacity to scaffold in late life is likely shaped by cumulative histories of cognitive demand, social engagement, stress regulation, physical activity, and environmental complexity.

The present framework does not argue that cognitive reserve and resilience are identical. Rather, it proposes that several adaptive processes identified across species (regulation, flexibility, social buffering, environmental engagement, and compensatory reorganization) may contribute to the development or expression of cognitive reserve in humans. When preserved cognition is observed despite age-related brain change or neuropathology, that phenotype can be described as cognitive resilience or as better-than-expected cognitive functioning, depending on the adopted nomenclature. Animal models can help isolate candidate mechanisms under controlled conditions, whereas human aging studies can test whether those mechanisms explain variability in cognitive outcomes beyond established reserve proxies and measures of neural burden.

### 5.2 Reserve proxies as behavioral enrichment and the confound problem

The empirical literature on cognitive reserve has relied heavily on proxy measures, including education, occupational complexity, bilingualism, social engagement, and cognitively stimulating leisure activities. These proxies are theoretically useful because they capture long-term exposure to environments that require learning, problem solving, communication, executive control, and sustained adaptation. They are also supported by epidemiological evidence showing that complex patterns of mental activity across early, mid-, and late-life are associated with reduced dementia incidence (Valenzuela and Sachdev, 2006), and that leisure activities are associated with lower dementia risk in older adults (Verghese et al., 2003). However, in the present framework, these proxies are not interpreted merely as demographic indicators. They are treated as indirect markers of repeated behavioral enrichment across the lifespan.

This interpretation helps clarify why traditional reserve proxies may be protective. Education not only transmits knowledge; it also fosters effortful learning, sustained attention, error correction, strategy use, rule-governed behavior, and persistence under evaluative demands. Occupational complexity requires repeated problem solving, planning, decision-making, inhibition of ineffective responses, and adaptation to changing contingencies. Bilingualism involves the continuous management of competing linguistic representations and has been proposed as one pathway to cognitive reserve through sustained executive and attentional control demands (Bialystok, 2021; Bialystok et al., 2012). Social engagement requires perspective-taking, communication, emotional regulation, coordination, and flexible adjustment to interpersonal contexts, and active social integration has been associated with lower dementia risk in late life (Fratiglioni et al., 2004). From this perspective, reserve proxies may function partly as forms of behavioral enrichment.

The analogy with animal enrichment is useful but should be made cautiously. In rodents, environmental enrichment typically combines novelty, spatial complexity, social housing, physical activity, and opportunities for exploration. These conditions influence hippocampal neurogenesis, synaptic plasticity, learning, and behavioral variability (Kempermann, 2019). Human education, work, bilingual language use, social engagement, and leisure activities are not direct equivalents of rodent enrichment. Nevertheless, both literatures converge on the idea that repeated engagement with complex environments can shape the biological and behavioral systems that support adaptive cognition.

This interpretation also fits more recent human evidence. For example, mid-life intellectual, physical, and social activities have been shown to contribute to late-life cognitive reserve independently of education, occupation, and late-life activities, and to moderate the relation between brain structure and cognitive performance (Chan et al., 2018). Such findings are important because they move beyond static early-life proxies and suggest that reserve-relevant enrichment may continue across adulthood.

At the same time, the reserve-proxy literature carries important methodological limitations. Education, occupational complexity, bilingualism, leisure activity, and social engagement are coarse indicators. They combine multiple processes that are difficult to separate: true capacity-building through enriched experience, genetic and cognitive endowment, socioeconomic advantage, access to health care, reduced exposure to chronic stress, and differential opportunities for engagement. Higher-ability individuals may be more likely to enter demanding educational and occupational environments. Individuals with greater socioeconomic resources may experience less cumulative allostatic load and better health across the lifespan. Conversely, early preclinical cognitive decline may reduce engagement in social, occupational, or leisure activities before formal cognitive assessment, creating the possibility of reverse causation.

These limitations do not invalidate the cognitive reserve literature, but they do constrain causal interpretation. Reserve is a hypothetical construct that cannot be measured directly, and proxy variables often stand in for mechanisms that remain underspecified (Jones et al., 2011). Similarly, critiques of the “use it or lose it” hypothesis have emphasized that associations between mental activity and cognitive aging may reflect selection effects, prior cognitive ability, or other confounding factors rather than direct causal protection alone (Salthouse, 2006). The present framework, therefore, does not claim that education, occupation, bilingualism, or social engagement automatically produce reserve. Rather, it asks what behavioral and biological processes these experiences may cultivate.

This reframing turns a broad epidemiological association into a more specific mechanistic prediction. If reserve proxies are protective because they reflect behavioral enrichment, then their effects should be partly mediated by measurable adaptive repertoires: coping flexibility, strategy shifting, sustained engagement, stress regulation, social problem solving, and compensatory reorganization under challenge. In other words, the key question is not simply whether education or occupational complexity predicts better late-life cognition. The more precise question is whether the behavioral capacities developed through demanding environments explain why some individuals maintain cognitive function despite neural aging or pathology.

This prediction requires research designs that go beyond proxy-outcome associations. Future studies should measure reserve proxies, behavioral resilience indicators, stress-regulatory markers, and neural burden within the same longitudinal framework. Such designs would allow researchers to test whether coping flexibility, adaptive strategy use, social regulation, or compensatory neural recruitment mediate the association between enriched life experience and cognitive outcomes. This would move the field from asking whether proxy variables “protect” the brain in a general sense toward identifying the behavioral and biological pathways through which reserve is built and expressed.

### 5.3 The integrative claim

The integrative claim of this section is not that cognitive reserve should be redefined as a behavioral repertoire. Cognitive reserve is retained as an established reserve-related property or capacity whose mechanisms remain under investigation. The present proposal is that observable behavioral and regulatory repertoires may constitute mechanisms through which reserve is developed, expressed, or deployed when demands increase. Flexible strategy use, compensatory reorganization, sustained engagement, stress recovery, and social resource mobilization are therefore treated as candidate process variables, not as interchangeable definitions or direct cumulative measures of cognitive reserve (Scheffer et al., 2018; Stern et al., 2020).

This reframing does not reduce cognitive reserve to psychological resilience, nor does it project the human concept of cognitive reserve onto animals. Rather, it proposes that both constructs can be understood as related expressions of a broader adaptive principle: organisms with richer, more flexible behavioral and regulatory repertoires are better able to maintain, recover, or reorganize function across a wider range of biological and environmental challenges. In animal models, this principle is expressed through maintained exploration, flexible responding, recovery after stress, and preservation of social or reward-related behavior. In human cognitive aging, it is expressed as better-than-expected cognitive performance despite brain aging, injury, or neuropathology.

This interpretation aligns with contemporary cognitive aging frameworks that distinguish among brain reserve, brain maintenance, cognitive reserve, and compensation, while recognizing that these processes often interact (Cabeza et al., 2018; Stern et al., 2020). It also extends those frameworks by specifying a developmental and behavioral pathway through which reserve may be built. The experiences typically treated as reserve proxies, education, occupational complexity, bilingualism, social engagement, leisure activity, and physical activity, may not protect cognition simply because they represent demographic advantage or accumulated stimulation. They may also repeatedly exercise the adaptive capacities that later allow individuals to regulate, compensate, and remain engaged under neural challenge.

The central claim, therefore, is not that resilience and cognitive reserve are identical. It is that cognitive reserve may represent a human, late-life expression of adaptive resilience mechanisms that are more broadly observable across species. Animal research helps isolate the biological and behavioral mechanisms through which challenge, enrichment, social context, and controllability shape adaptation. Human aging research shows that similar adaptive capacities may become clinically evident when individuals experience age-related brain changes or neuropathology. The value of the proposed framework lies in connecting these levels of analysis without collapsing one into the other.

### 5.4 Disconfirming evidence and boundary conditions

A theoretically responsible framework must also identify the evidence that could limit, complicate, or disconfirm its claims. The resilience-reserve bridge proposed here is not intended as a universal explanation for all forms of cognitive preservation, all forms of adversity, or all neuropathological conditions. Its usefulness depends on several boundary conditions.

First, cognitive reserve effects may be pathology-dependent. The reserve literature has been developed most extensively in Alzheimer's disease and Alzheimer-related pathological change, where education, occupational complexity, and other reserve proxies have been repeatedly studied in relation to amyloid, tau, neurodegeneration, and clinical outcomes (Stern, 2012; Stern et al., 2020). Recent biomarker studies continue to show that reserve-related variables can modify the relationship between Alzheimer-type pathology and cognitive decline, although the strength and direction of these associations may depend on pathology stage, biomarker profile, and cognitive domain (Bocancea et al., 2023; McKenzie et al., 2023). This suggests that reserve is not a general shield against all neural damage. It may be more effective when the neural systems required for compensation, especially frontal-executive and network-level control systems, remain sufficiently intact to support adaptive reorganization.

Second, reserve may be expressed differently across dementia syndromes. In frontotemporal dementia, for example, reserve-related mechanisms have been studied, but the relevant clinical outcomes may involve behavioral and social functioning as much as traditional cognitive performance. Studies of granulin-related frontotemporal dementia suggest that cognitive reserve can modulate functional connectivity even in genetic forms of the disease (Premi et al., 2013). More recent work has proposed the related concept of behavioral reserve in behavioral-variant frontotemporal dementia, emphasizing that individuals may differ in behavioral symptoms despite comparable neuroimaging burden (Kim et al., 2022). These findings do not contradict the present framework, but they do refine it. If the affected neural systems differ across diseases, then the form of reserve may also differ. In Alzheimer's disease, reserve may appear primarily as preserved memory or executive function. In frontotemporal disorders, it may appear as relative preservation of behavioral regulation, social functioning, or daily adaptive behavior.

Third, enrichment effects have limits. Animal studies show that environmental enrichment can influence neuroplasticity, learning, exploratory behavior, and stress-related outcomes, but these effects should not be interpreted as unlimited protection against severe or progressive pathology (Kempermann, 2019; Nithianantharajah and Hannan, 2006). Enrichment may delay functional impairment, strengthen compensatory capacity, or improve adaptation under moderate biological burden, but it does not necessarily prevent neuropathology itself. This distinction is essential for the present framework. The claim is not that resilience-related behavioral repertoires prevent neural aging or disease. The claim is that they may extend the window during which function can be maintained despite increasing burden.

Fourth, the benefits of challenge are conditional. The stress inoculation literature does not support the simplistic conclusion that adversity is good for development. In nonhuman primates and other animal models, resilience-building effects are most plausible when challenges are moderate, time-limited, controllable, and embedded in a context that allows recovery and social buffering (Lyons and Parker, 2007; Parker and Maestripieri, 2011). Severe, chronic, unpredictable, or uncontrollable adversity is more likely to increase allostatic load and compromise regulation than to strengthen resilience (Daskalakis et al., 2013; McEwen, 1998). This boundary condition is central. The proposed framework does not argue that adverse experience generally builds cognitive reserve. It argues that manageable challenges, combined with opportunities for control, learning, support, and recovery, may strengthen the regulatory and behavioral capacities that later contribute to reserve.

Fifth, reserve proxies remain methodologically imperfect. Education, occupation, bilingualism, social engagement, and leisure activity are indirect indicators. They may reflect true behavioral enrichment, but they also capture socioeconomic advantage, baseline cognitive ability, genetic influences, access to resources, and selection into stimulating environments. Early preclinical decline may also reduce engagement before formal diagnosis, creating the possibility of reverse causation. These limitations have been repeatedly noted in the reserve literature and remain unresolved by the present framework (Jones et al., 2011; Salthouse, 2006). For this reason, the framework should not be read as claiming that reserve proxies are causal mechanisms. Rather, it proposes that they may be imperfect markers of underlying behavioral and regulatory repertoires that must be measured more directly.

These boundary conditions make the framework more testable. The resilience-reserve connection should be strongest when: individuals face neuropathological burdens for which compensatory systems remain at least partially available; enrichment occurs across the life course rather than only after decline is established; challenges are manageable rather than chronically overwhelming; and behavioral resilience indicators are measured independently from traditional reserve proxies.

The framework would be weakened if behavioral resilience indicators did not predict cognitive trajectories after adjustment for education, occupational complexity, socioeconomic status, baseline cognition, and structural brain measures. It would also be weakened if the effects of animal enrichment on preserved cognitive function were not mediated by changes in behavioral flexibility, stress regulation, or compensatory neural recruitment. Similarly, it would be weakened if human neuroimaging studies showed no association between coping flexibility, stress-regulation history, and compensatory prefrontal or network-level recruitment. These possible null findings are important because they clarify that the present proposal is not only interpretive. It makes a specific empirical prediction: traditional reserve proxies should exert part of their protective effect through measurable behavioral and regulatory repertoires.

## 6 An integrative framework: five levels of adaptive resilience

The framework proposed here organizes resilience across five interacting levels: biological regulation, behavioral flexibility, social regulation, environmental enrichment, and functional preservation. These levels are not strictly hierarchical. They are mutually reinforcing systems that operate across development and across species. The purpose of the model is not to present a fully established causal pathway, but to offer an organizational scaffold that can generate testable hypotheses about how resilience-related processes may become expressed in humans as cognitive reserve.

The proposed pathway can be summarized as follows. Early biological regulation, shaped by caregiving, stress exposure, genetic vulnerability, and developmental context, influences the sensitivity of the HPA axis, autonomic, immune, hippocampal, and prefrontal systems. These regulatory systems help determine the organism's capacity for behavioral flexibility: the ability to shift strategies, inhibit ineffective responses, explore alternatives, and recover after disruption. Social regulation buffers stress responses and provides external scaffolding for coping, problem solving, and emotional regulation. Environmental enrichment expands behavioral repertoires and stimulates neural plasticity, particularly in hippocampal and prefrontal systems. Together, these processes converge on functional preservation: the ability to maintain, recover, or reorganize adaptive behavior in response to biological or environmental challenges.

This pathway remains explicitly provisional. It should be understood as a falsifiable proposal rather than an established causal model. Its value lies in specifying measurable levels and candidate mechanisms: stress-response regulation, behavioral flexibility, social buffering, environmental engagement, and preserved function. These mechanisms provide a bridge between animal resilience research and human cognitive reserve without assuming that the constructs are identical.

### 6.1 Level 1: biological regulation

The foundation of resilience is efficient biological regulation. This includes neuroendocrine reactivity and recovery, particularly HPA-axis function; autonomic regulation; immune and inflammatory modulation; genetic and epigenetic influences on stress systems; and neural plasticity supporting learning, adaptation, and recovery. McEwen's (1998) allostasis framework is central here. From this perspective, resilience does not require the absence of stress activation. Rather, it depends on the appropriate calibration, containment, and termination of stress responses over time.

Biological regulation is shaped by early experience. In rodent models, naturally occurring variation in maternal care has been associated with stable individual differences in HPA-axis reactivity, hippocampal development, and stress-related behavior (Meaney, 2001). Epigenetic studies further show that maternal behavior can alter hippocampal glucocorticoid receptor expression through DNA methylation-related mechanisms, providing a pathway through which early caregiving may become biologically embedded (Weaver et al., 2004). In this framework, early adversity may compromise biological regulation, whereas supportive caregiving and manageable, controllable challenge may help calibrate stress systems in ways that support later adaptation.

Neuroautonomic Adaptive Resilience (NAR) provides a more specific example of a dynamic resilience mechanism within this biological-regulation domain. Fusi et al. (2026) conceptualize NAR as a self-regulatory mechanism involving flexible coordination between central and autonomic nervous-system processes in response to cognitive and emotional stressors. Within the present framework, NAR is therefore not treated as a form of cognitive reserve or as a reserve mechanism itself. Rather, it represents an adaptive regulatory process that may interact with cognitive reserve and with maintenance- and compensation-related mechanisms in shaping functional responses to challenge. This positioning preserves the distinction between dynamic resilience mechanisms and reserve-related constructs while allowing their interaction to be examined empirically.

### 6.2 Level 2: behavioral flexibility

Behavioral flexibility is the most directly observable expression of resilience and the level at which cross-species comparison is most tractable. It refers to the capacity to shift strategies, inhibit ineffective responses, explore alternatives, update behavior when contingencies change, and reorganize action after disruption. In animals, behavioral flexibility may be inferred from maintained exploration, recovery of reward-seeking, social approach, reversal learning, strategy shifting, and persistence after stress exposure. In humans, it is expressed through coping flexibility, adaptive problem solving, compensatory strategy use, emotional regulation, and the reorganization of routines when previous strategies fail.

This level is central because it links biological regulation to functional outcome. An organism with efficient stress recovery may be better able to explore, learn, and adapt after a challenge. Conversely, chronic dysregulation may narrow behavioral repertoires and promote avoidance, rigidity, or disengagement. Behavioral flexibility depends on prefrontal, striatal, and hippocampal systems that are sensitive to stress history, enrichment, and aging. It is therefore a plausible mediating mechanism between early experience and later resilience outcomes, including cognitive reserve.

### 6.3 Level 3: social regulation

Social relationships exert powerful effects on stress regulation and adaptive behavior. Across animal and human studies, affiliative social contact can buffer HPA-axis responses to threat or challenge, although the magnitude of this effect depends on developmental timing, relationship quality, prior experience, and species-specific social organization (Hennessy et al., 2009; Hostinar et al., 2014). This makes social regulation more than an external support. It is part of the biological and behavioral system through which resilience is built and expressed.

In developmental science, supportive relationships are among the most consistent predictors of resilient outcomes in children and adolescents (Masten, 2001; Southwick et al., 2014). In dogs, positive human–dog relationships, predictability, social support, and opportunities for control have also been discussed as protective factors for behavioral wellbeing (Tiira, 2019). Across species, social regulation can reduce stress activation, increase predictability, support exploration, and provide external scaffolding when internal regulatory resources are limited.

In cognitive aging, social engagement is often treated as a reserve proxy. The present framework suggests that its protective role may operate through at least two pathways. First, social interaction exercises cognitive capacities such as attention, memory, communication, perspective-taking, inhibition, and emotional regulation. Second, social relationships may reduce cumulative allostatic load by buffering stress responses and supporting recovery. Social engagement may therefore contribute to cognitive reserve both by enriching cognition and by protecting the biological systems that sustain adaptive function.

### 6.4 Level 4: environmental enrichment

Environmental enrichment refers to sustained engagement with complex, variable, and stimulating conditions. In rodents, enrichment typically includes novel objects, spatial complexity, social housing, and opportunities for physical activity. In humans, analogous enrichment may include education, occupational complexity, bilingualism, physical activity, leisure engagement, social participation, and cognitively demanding environments. The parallel is not exact, but it is conceptually useful: in both cases, enriched environments expand behavioral repertoires and stimulate neural systems involved in learning, exploration, and adaptation.

Rodent enrichment studies have shown robust effects on neuroplasticity, including changes in adult neurogenesis, synaptic plasticity, exploratory behavior, and cognitive performance (Nithianantharajah and Hannan, 2006). Kempermann (2019) later argued that environmental enrichment and adult neurogenesis provide a useful framework for understanding individual differences in brain plasticity and reserve-relevant adaptation. In humans, reserve proxies such as education and occupational complexity may function, at least in part, as forms of behavioral enrichment that repeatedly exercise strategy use, problem solving, self-regulation, and compensatory flexibility.

However, enrichment should not be interpreted as simple stimulation or as guaranteed protection. Its effects are likely conditional on timing, intensity, accessibility, individual vulnerability, and the presence of supportive social and biological contexts. Enrichment is most relevant to the present framework when it creates opportunities for active engagement, learning, exploration, and adaptive control rather than passive exposure to complexity.

### 6.5 Level 5: functional preservation and recovery

The final level represents the adaptive system's functional outcome: the maintenance, recovery, or reorganization of behavior after adversity, stress, deprivation, disease, aging, or other biological or environmental challenge. In nonhuman animals, this outcome can be assessed through observable patterns such as preserved or recovered exploration, social approach, reward seeking, learning, cognitive performance, and adaptive routines following perturbation. In humans, relevant outcomes may include preserved cognitive performance, functional independence, daily adaptive behavior, quality of life, recovery after disruption, and better-than-expected functioning relative to the degree of neural or clinical burden.

This distinction is particularly important for positioning cognitive reserve within the present framework. Cognitive reserve should not be equated with Level 5 functional preservation itself. Contemporary reserve models describe cognitive reserve as a capacity that may influence how effectively individuals cope with age-related brain change, injury, or neuropathology (Stern et al., 2020). In human cognitive aging, better-than-expected cognitive performance despite such neural burden is therefore more appropriately treated as a cognitive resilience phenotype, or as the functional outcome that cognitive reserve is hypothesized to support (Kremen et al., 2022; Stern et al., 2020). Cognitive reserve is thus one potential contributor to preserved cognitive functioning rather than the preserved functioning itself.

This distinction also recognizes that cognitive reserve is unlikely to operate in isolation. Brain reserve, brain maintenance, compensation, and other domain-specific reserve mechanisms may contribute to functional preservation through partly distinct but interacting pathways. Brain reserve may provide greater neurobiological capacity, brain maintenance may reduce the degree of age-related neural deterioration, and compensation may permit the recruitment of alternative neural or cognitive strategies when primary systems become insufficient. Cognitive reserve may influence the flexible or efficient use of available resources under conditions of neural challenge. Consequently, similar functional outcomes may arise from different combinations of reserve, maintenance, compensation, and adaptive processes.

For example, an older adult who continues to reason, communicate, plan, and function independently despite substantial Alzheimer-related neuropathology demonstrates resilience at the functional level. The preserved performance is the outcome to be explained rather than direct evidence that cognitive reserve itself constitutes that outcome. Cognitive reserve may contribute to this preservation, but so may relatively maintained brain integrity, compensatory recruitment, adaptive strategy use, social resources, and other regulatory processes. Framing Level 5 in this way therefore avoids inferring a single mechanism from successful functioning and instead allows the relative contribution of different reserve-related pathways to be examined empirically.

A comparable logic applies to animal research without projecting human clinical constructs directly onto nonhuman species. An animal that continues to explore, learn, seek reward, interact socially, or reorganize behavior after exposure to stress or biological challenge demonstrates functional preservation or recovery in a species-appropriate form. Such outcomes should not be interpreted as evidence that the animal possesses cognitive reserve in the human clinical sense. Rather, they provide experimentally accessible manifestations of the broader adaptive problem addressed by the framework and allow candidate mechanisms of preservation, recovery, and reorganization to be investigated under controlled conditions.

The proposed framework therefore links Levels 1–4 to Level 5 while maintaining a clear distinction between adaptive processes and functional outcomes. Biological regulation may support effective physiological recovery and maintain conditions necessary for subsequent adaptation. Behavioral flexibility permits strategy shifting, inhibition of ineffective responses, and adjustment to changing contingencies. Social regulation can buffer stress and provide external scaffolding for coping and problem solving. Environmental engagement and enrichment may expand behavioral repertoires and repeatedly recruit neural systems involved in learning and plasticity. In humans, these processes may contribute to the development or expression of cognitive reserve and other reserve-related capacities; their functional consequences become observable at Level 5 through the degree to which cognition and adaptive behavior are maintained, recovered, or reorganized under increasing biological or environmental demands.

Level 5 should therefore be understood as the outcome level of the framework, not as cognitive reserve itself. This distinction preserves the conceptual separation between reserve-related mechanisms and cognitive resilience while retaining the central cross-species argument: organisms may rely on different combinations of biological, behavioral, social, and environmental processes to preserve or reorganize function under conditions that impair others.

## 7 Implications for cognitive aging and neuropsychology

The framework proposed in this manuscript has several implications for theory, research, and practice in cognitive aging and neuropsychology. Its central contribution is not to recast cognitive reserve as a dynamic or lifespan-based construct, because contemporary reserve models already recognize active mechanisms and the influence of genetic and environmental factors operating across the life course (Stern et al., 2020, 2023). Rather, the framework adds a cross-species process layer that connects life-course exposures with potentially measurable biological, behavioral, social, and environmental mechanisms and, ultimately, with functional outcomes under neural challenge. In this formulation, cognitive reserve remains an established human reserve construct; the proposed adaptive processes are candidate contributors to its development or expression rather than alternative definitions of cognitive reserve itself.

First, the framework encourages a clearer distinction among reserve-related exposures and proxies, process measures, and neural or functional outcomes. Cognitive reserve has frequently been investigated indirectly through variables such as education, occupational complexity, intellectual engagement, and other life-experience indicators (Jones et al., 2011; Stern et al., 2020). These measures are valuable because they capture aspects of cumulative experience associated with differences in later cognitive functioning, but they do not directly identify the mechanisms through which those experiences may influence adaptation to neural challenge. The present framework therefore proposes complementing traditional proxies with measures of the processes that may mediate or express reserve-related capacity.

This distinction is particularly important for neuropsychological research. Education, occupational complexity, sustained cognitive engagement, social participation, and similar variables can be treated as life-course exposures or proxies that may contribute to reserve. By contrast, strategy use, error monitoring, flexible adaptation, response to feedback, persistence after difficulty, compensatory routines, and adaptive help-seeking should not be interpreted as cumulative measures of cognitive reserve. They are better understood as current process or expression measures that may reveal how an individual responds when cognitive demands increase. Similarly, structural brain measures should not be described as direct measures of cognitive reserve. Depending on the design and theoretical framework, they may characterize neural status or brain reserve, and longitudinal change in such measures may contribute to the study of brain maintenance (Stern et al., 2020, 2023). The empirical prediction of the present framework is therefore not that process measures should replace established reserve proxies, but that they may explain additional variance in cognitive resilience after accounting for life-course exposures, baseline cognitive ability, and neural burden.

Second, the framework clarifies the relationship between psychological resilience and cognitive reserve without treating them as interchangeable constructs. Both literatures address variation in functioning under conditions of challenge, but they begin from different problems and use different operational definitions. Psychological resilience research generally asks how functioning is maintained, recovered, or reorganized following adversity or stress. Cognitive reserve research asks how individuals differ in their ability to cope with age-related brain changes, injury, or neuropathology (Stern et al., 2020). Cognitive resilience, in turn, refers more directly to the observed preservation of cognitive functioning relative to the degree of adverse brain factors (Kremen et al., 2022). The conceptual bridge proposed here lies not in equating these constructs but in identifying potentially shared processes—including regulation, behavioral flexibility, compensatory responding, social buffering, and sustained environmental engagement—that may contribute to adaptive functioning across these different contexts.

Greater dialogue between these research traditions may therefore be useful in both directions. Resilience research provides experimental and developmental models for studying how regulation, recovery, and behavioral adaptation unfold over time and under changing environmental demands. Cognitive reserve research provides a well-developed framework for examining individual differences in cognition in relation to brain aging and neuropathology. Connecting the two may help generate more precise hypotheses about the processes through which life-course experiences become relevant to later cognitive functioning, while maintaining the conceptual boundaries among cognitive reserve, cognitive resilience, brain reserve, brain maintenance, and compensation.

Third, the framework adopts a lifespan perspective that is consistent with, rather than corrective of, contemporary reserve and resilience theory. Cognitive reserve is already understood to be influenced by experiences and exposures operating at multiple points across life, and recent frameworks increasingly emphasize the importance of life-course diversity, environmental opportunity, and cumulative biological and social context in understanding brain resilience (Stern et al., 2023; Reuter-Lorenz and Park, 2024; Rajah et al., 2026; Udeh-Momoh et al., 2025). Developmental resilience theory similarly conceptualizes adaptive functioning as emerging through interactions among biological regulation, caregiving, learning, social relationships, and environmental conditions over time (Masten, 2001; Southwick et al., 2014). The contribution of the present framework is therefore not the proposition that reserve begins before old age, but the specification of candidate processes through which life-course exposures may become biologically and behaviorally instantiated.

From this perspective, caregiving and early social experience may contribute to the calibration of biological stress-regulatory systems; educational and occupational demands may repeatedly engage learning, problem solving, and behavioral flexibility; social relationships may provide both cognitive engagement and physiological buffering; physical activity may influence neural and systemic health; and continued interaction with complex environments may provide opportunities for strategy development and adaptive responding. These influences should not be assumed to produce reserve automatically or uniformly. Their effects are likely to depend on timing, intensity, accessibility, socioeconomic context, prior ability, biological vulnerability, and the nature of the challenges encountered. A lifespan formulation therefore emphasizes cumulative and interacting pathways rather than a simple additive model of reserve acquisition.

Within this lifespan perspective, cognitive reserve may also interact with other domain-specific reserve constructs that have been proposed to account for individual differences in adaptation across distinct functional systems. Affective reserve has been proposed to describe capacities relevant to the regulation of affective functioning under adversity, pathology, or aging (Di Rosa, 2024), while motor reserve addresses individual differences in the preservation of motor function despite disease-related neural burden (Giustiniani and Quartarone, 2024). Behavioral reserve has likewise been proposed in behavioral-variant frontotemporal dementia to characterize variation in behavioral expression relative to neurodegenerative burden (Kim et al., 2022). These constructs should not be treated as interchangeable with cognitive reserve, because they concern different functional domains and may depend on partly distinct mechanisms. Nevertheless, they may develop across the lifespan and interact with cognitive reserve, brain maintenance, compensation, and one another in shaping trajectories of aging. Their inclusion therefore broadens the present framework while preserving cognitive reserve as its primary translational focus in cognitive aging.

Fourth, the framework encourages a more process-oriented approach to neuropsychological research and interpretation. Conventional cognitive scores remain essential, but similar final scores can sometimes arise through different patterns of performance. One individual may solve a task efficiently using a familiar strategy, whereas another may require additional time, generate alternative approaches, benefit from cueing, or recruit compensatory routines to achieve a comparable result. These differences do not constitute direct measures of cognitive reserve, nor should they automatically be interpreted as evidence of greater or lesser reserve. They are, however, potentially informative process variables that may reveal how adaptive capacity is expressed during cognitive challenge.

This perspective suggests that neuropsychological research could complement endpoint scores with carefully operationalized indicators such as strategy generation and switching, response to corrective feedback, adaptation after errors, susceptibility to fatigue or increasing task demand, response to cueing, and use of external compensatory supports. Longitudinal designs would be particularly valuable because they could test whether these process characteristics predict subsequent cognitive or functional outcomes independently of education, occupation, baseline cognitive ability, and neural burden. Such an approach would move the framework from conceptual description toward direct empirical evaluation.

Fifth, the framework highlights the translational value of animal models while preserving the limits of cross-species inference. Animal models cannot reproduce human language, autobiographical memory, culturally mediated meaning, subjective appraisal, or the full clinical construct of cognitive reserve. They can, however, isolate candidate mechanisms that are difficult or impossible to manipulate experimentally in humans. Environmental enrichment paradigms, maternal-care models, stress-controllability designs, and stress-inoculation studies provide experimental access to processes such as stress regulation, behavioral flexibility, social buffering, learning, and adaptive engagement. Model systems have also been explicitly proposed as useful tools for the mechanistic study of reserve-related cognitive aging because they permit longitudinal investigation and experimental manipulation of biological and environmental variables (McQuail et al., 2020).

The translational logic is therefore mechanistic rather than categorical. An animal that preserves exploration, learning, reward seeking, or social engagement following a challenge is not demonstrating cognitive reserve in the human clinical sense. Instead, the animal model allows investigators to examine biological and behavioral processes that may have homologous or analogous relevance to the mechanisms supporting preserved cognition in humans. This distinction provides a more conservative basis for cross-species inference and makes the proposed framework empirically testable without assuming equivalence between animal resilience and human reserve constructs.

Finally, the framework has implications for intervention research in cognitive aging. The present model does not imply that cognitive reserve itself is simply a behavioral repertoire that can be directly trained. Rather, it suggests that some of the processes and exposures associated with the development or expression of reserve-related capacities may be modifiable. Interventions might therefore examine combinations of cognitive engagement, flexible problem solving, physical activity, stress-regulation strategies, social participation, and compensatory skill use rather than focusing exclusively on cognitive stimulation. This proposal is compatible with cognitive-neuroscience models that distinguish maintenance, reserve, and compensation while emphasizing adaptive neural recruitment and life-course plasticity (Cabeza et al., 2018; Reuter-Lorenz and Park, 2014, 2024).

The practical implication is consequently more specific than treating reserve as something that can simply be increased through training. Future intervention studies should determine whether particular experiences or behavioral and regulatory processes modify the relationship between neural burden and functional outcome, whether their effects operate through brain maintenance, cognitive reserve, compensation, or other mechanisms, and whether those effects generalize across cognitive domains and clinical populations. Framed in this way, the proposed model offers a set of measurable pathways through which the broader relationship among life experience, adaptive regulation, reserve-related mechanisms, and cognitive resilience can be tested across the lifespan.

## 8 Limitations

Several limitations of the proposed framework must be acknowledged. First, species differences are real and substantial. Human resilience encompasses dimensions that cannot be directly translated to nonhuman animals, such as language, conscious appraisal, autobiographical memory, self-narrative, cultural meaning-making, and reflective identity. The present framework does not claim that animal and human resilience are identical. Rather, it argues that they may share adaptive logic and partially overlapping biological and behavioral mechanisms. This distinction is essential. Without it, comparative research risks two opposite errors: naïve anthropomorphism when interpreting animal behavior, and naïve biologism when interpreting human psychological adaptation.

Second, cognitive reserve remains a human clinical and cognitive-aging construct. It was developed to explain why some individuals maintain better cognitive performance than expected despite brain aging, injury, or neuropathology (Stern, 2012; Stern et al., 2020). For this reason, cognitive reserve should not be projected directly onto animals. The argument advanced here is more limited: the behavioral and regulatory processes that support cognitive reserve in humans may have evolutionary and comparative precursors. Animals do not possess cognitive reserve in the full clinical, autobiographical, or cultural sense. However, animal models can help clarify mechanisms of behavioral flexibility, stress regulation, compensation, and functional preservation that may later appear in humans as reserve-related resilience.

Third, adversity is not inherently beneficial. The stress inoculation literature suggests that moderate, controllable, and socially buffered challenges may promote regulatory development under specific conditions (Lyons and Parker, 2007; Parker and Maestripieri, 2011). This finding should not be generalized into the claim that adversity itself produces resilience. Severe, chronic, unpredictable, uncontrollable, or socially isolated adversity is more likely to increase allostatic load, disrupt regulation, and impair development (Daskalakis et al., 2013; McEwen, 1998). The protective effects of manageable challenge are therefore conditional. They should be interpreted as evidence about the boundaries of developmental plasticity, not as a general endorsement of adversity as a developmental asset.

Fourth, the proposed five-level model is conceptual. Each level of the framework, biological regulation, behavioral flexibility, social regulation, environmental enrichment, and functional preservation, is supported by substantial empirical literature. However, the specific pathways linking these levels remain hypothetical. For example, the proposed pathway from early social regulation to later functional preservation through stress buffering, behavioral flexibility, and prefrontal-hippocampal plasticity has not yet been directly tested as an integrated longitudinal model. The framework should therefore be understood as a theory-generating structure rather than as an established causal account.

Fifth, reserve proxy research has important methodological limitations. Education, occupational complexity, bilingualism, leisure activity, and social engagement are useful indicators, but they are also coarse and confounded measures. They may reflect genuine enrichment, but they also capture socioeconomic status, baseline cognitive ability, genetic influences, access to health care, health behaviors, selection effects, and opportunities for engagement. In addition, declining cognition may reduce participation in complex activities before formal diagnosis, creating the possibility of reverse causation. These concerns have been noted in critiques of cognitive reserve measurement and of the “use it or lose it” hypothesis (Jones et al., 2011; Salthouse, 2006). Stronger evidence for the proposed resilience-reserve connection will require prospective studies that measure behavioral repertoires, biological regulation, neural burden, and cognitive outcomes over time.

A final limitation concerns operationalization. If the resilience-reserve interface is to become empirically useful, resilience cannot remain only a broad explanatory label. It must be measured through observable indicators across multiple levels. Candidate biological measures include cortisol recovery, diurnal cortisol slope, inflammatory markers, autonomic flexibility, glucocorticoid sensitivity, and prefrontal-hippocampal network integrity. Candidate behavioral measures include reversal learning, strategy shifting, error correction, persistence after failed attempts, coping flexibility, adaptive help-seeking, and compensatory strategy use. Candidate social measures include relationship quality, perceived support, social integration, and the availability of external scaffolding during cognitive or emotional challenge. Candidate reserve-related outcomes include better-than-expected cognitive performance given neuropathology, slower functional decline, preserved daily functioning, and maintained adaptive behavior under increasing neural burden.

These limitations do not weaken the framework; they define its scope. The framework is not a claim that resilience and cognitive reserve are the same construct, that adversity is beneficial, or that animal models can fully explain human aging. Rather, it is a proposal that adaptive regulation, behavioral flexibility, social buffering, and environmental enrichment may help explain why some organisms and, in humans, some aging individuals maintain function under conditions that impair others. Its value will depend on whether these proposed mechanisms can be measured directly and tested prospectively.

## 9 Conclusion

The question that motivates this manuscript is simple but far-reaching: why do some organisms maintain adaptive functioning under conditions that impair others? This question has been examined for decades across animal stress research, developmental psychology, clinical neuropsychology, and cognitive aging. Yet these fields have often addressed the same adaptive problem using different languages, different methods, and largely separate literatures. This manuscript has argued that these domains can be brought into closer conversation through a shared focus on regulation, flexibility, enrichment, and functional preservation.

The central claim is not that animal resilience and human cognitive reserve are the same construct. They are not. Cognitive reserve is a human clinical and cognitive-aging concept, rooted in the observation that some individuals function better than expected despite brain aging, injury, or neuropathology. Animal resilience models, by contrast, examine observable behavioral and biological adaptations following stress, adversity, or environmental challenges. The proposed bridge is therefore not one of identity, but of mechanism. Across species, adaptive functioning depends on partly shared systems: prefrontal executive control, hippocampal plasticity, HPA-axis regulation, social buffering, behavioral flexibility, and the capacity to reorganize behavior when prior strategies are no longer sufficient.

From this perspective, cognitive reserve can be understood as a human, late-life expression of broader adaptive resilience processes. It is not merely a passive neural buffer conferred by education, occupation, or intellectual activity. It may also reflect an active behavioral and regulatory repertoire built across the lifespan: the capacity to use strategies flexibly, compensate when primary systems weaken, regulate stress responses, mobilize social resources, remain engaged with demanding environments, and preserve function under increasing neural burden. The STAC-r model describes one neural expression of this process in aging adults: compensatory scaffolding in response to neural challenge. The present framework complements that account by asking how such compensatory capacity is developmentally and behaviorally built.

The framework also has limits, and those limits are important. Reserve effects are not uniform across all neuropathologies. Enrichment may delay functional impairment without preventing neuropathology itself. Stress inoculation is not a general argument in favor of adversity; it applies only to manageable, controllable, time-limited challenges embedded in conditions that allow recovery and social buffering. These boundary conditions do not weaken the framework. They make it more precise. A useful theory should not explain everything; it should specify when, how, and for whom its predictions are likely to hold.

The strongest version of the argument is therefore not that cognitive reserve is itself a species-specific form of adaptive resilience. Rather, adaptive resilience research identifies candidate biological, behavioral, and social processes that may contribute to the development and expression of cognitive reserve and to cognitive resilience under neural challenge. In this formulation, cognitive reserve remains a human reserve construct; preserved cognition despite neuropathology is the functional outcome to be explained; and animal models provide experimental leverage for isolating mechanisms such as stress regulation, behavioral flexibility, social buffering, environmental engagement, and compensatory reorganization. The proposed framework extends existing reserve models by adding a comparative and mechanistic level of analysis, not by replacing their definitions.

The practical implication is clear. If behavioral resilience repertoires contribute to cognitive reserve, then they should be measured directly. Coping flexibility, strategy diversity, adaptive error correction, persistence after difficulty, social resource mobilization, stress recovery, and compensatory behavior may add predictive value beyond education, occupation, cognitive test scores, and structural neuroimaging. This is a testable claim. Future longitudinal and translational studies can determine whether these behavioral and regulatory capacities explain part of the protective effect currently attributed to proxies of cognitive reserve. Testing that possibility is the next step.
